# Mental health and self-determination profiles of the diverse population of medical students in Malaysia during the COVID-19 pandemic

**DOI:** 10.1186/s40359-022-00759-y

**Published:** 2022-03-03

**Authors:** Jessica Grace Cockburn, Chee Yang Tan, Dawn Celine Siaw Chern Poh, Ding Jun Tan, Chan Choong Foong, Wei-Han Hong

**Affiliations:** 1 Medical Education and Research Development Unit, Faculty of Medicine, Universiti Malaya, 50603 Kuala Lumpur, Malaysia; 2 Society of Malaysia Medical Association Medical Students, Malaysia Medical Association, 53000 Kuala Lumpur, Malaysia; 3 Faculty of Medicine, Universiti Malaya, 50603 Kuala Lumpur, Malaysia; 4 Newcastle University Medicine Malaysia, 79200 Nusajaya, Johor Malaysia

**Keywords:** COVID-19, Self-determination, Diversity, Undergraduate medical education, Autonomy, Relatedness, Competence, Mental health, Well-being

## Abstract

**Introduction:**

Medical schools throughout the world were forced to modify their programming during the COVID-19 pandemic. In Malaysia, virtual learning plans were implemented for non-clinical programming, while clinical posting modifications were designed to meet local SOPs. The prolonged enforcement of these modifications to undergraduate medical education will have affected student experiences, including well-being. Since these feelings can relate to perceived relatedness, autonomy, and competence, it is important to identify any potential factors that may lead to reduced intrinsic motivation in students. It is also important to consider how demographic features may contribute to student perspectives, which can be studied using the unique diversity represented by Malaysian students.

**Methods:**

A quantitative survey was distributed to Malaysian medical students to assess their overall wellbeing, autonomy in educational decision making, student experiences, and position on changes to graduation timing. Intrinsic components were identified using Principal Component Analysis and were aligned with the three needs for self-determination, namely relatedness, autonomy, and competence. Finally, trends in responses for participants from various sub-populations were assessed using ANOVA testing.

**Results:**

Responses were collected from 442 students representing 23 accredited Malaysian medical schools. Upon validation and reliability testing, eight components were identified with themes relating to: mental health, social concerns, communication, timing of modifications, depth of learning, and student-centred learning. Of these, gender was related to mental health, student-centred learning, and delayed graduation, while stage was related to student-centred learning and delayed graduation in addition to concerns about depth of learning and timing of modifications. Interestingly, ethnicity was related to differences in opinions about delayed graduation and income was related to social concerns.

**Conclusion:**

The results of this study indicate that, while students were satisfied in general with the content and delivery of their programmes given the circumstances, there is evidence to suggest negative effects on emotional wellbeing and expression of student voice, due to the modifications that were made. Additionally, these feelings related to the three motivational needs, suggesting that students were experiencing a dampened motivational profile during the pandemic. Further, motivational profiles were distinct between student sub-groups, providing insight for developing appropriate and inclusive accommodations moving forward.

**Supplementary Information:**

The online version contains supplementary material available at 10.1186/s40359-022-00759-y.

## Background

In 2020, Malaysia, similar to other countries, was facing the challenges of a global pandemic due to the widespread transmission of the novel SARS-CoV-2 [1–7]. To mitigate this spread, the Malaysian government implemented strict lockdown protocols including the conversion of face-to-face educational programmes to virtual learning environments, including the non-clinical delivery of medical education. To meet these requirements, undergraduate medical programmes in Malaysia (MBBS) provided students with online-based courses, and modified student requirements in clinical postings. The national response in 2020 afforded relatively good control over the number of COVID-19 cases, though despite evolving restrictions, cases gradually rose through 2021, resulting in a corresponding burden on the healthcare system [8]. This also prolonged educational modifications as non-clinical teaching remained virtual and clinical postings were adapted depending on context.

There are currently 30 accredited undergraduate medical schools in Malaysia which offer five-year degrees. Each programme is divided into pre-clinical and clinical stages, with an increasing weightage of workplace-based learning during clinical stages. The student population reflects the diversity in Malaysia, which is predominantly comprised of three ethnic groups and includes a range of socioeconomic brackets. Additionally, resources are heterogeneously distributed across Malaysia, thus requiring institutions to individually facilitate programmes including the implementation of pandemic restrictions, while adhering to quality assurance guidelines set by governing bodies to maintain programme integrity [9].

The rapid transition to online learning in medical education globally required synchronous virtual learning environments, supported by various platforms and modifications specific to each institute’s resources and clinical requirements [10–15]. These virtual learning strategies have been validated as appropriate alternatives for educational delivery given that content delivery is sufficient and can additionally support flexible schedules, though most participants may prefer in-person sessions [16–20]. However, challenges may arise from technical resource availability or low levels of digital literacy [13, 20]. Additionally, clinical postings have required modifications to mitigate the risk of COVID-19 transmission [21]. The response of medical schools globally has been varied, but has included changes to graduation, reducing the amount of clinical practice time, and transition to virtual assessments, while trying to minimize the need to suspend placements [7, 22–24]. There has been an attempt to standardize these actions in the medical education community to maintain quality as the pandemic continues and to prepare for future disruptions [7, 23, 25, 26].

It is important to understand the impact of pandemic-related modifications on student learning experiences and wellbeing. This is particularly true in Malaysia where the fluctuating COVID-19 situation has led to both long-term reliance on virtual learning and frequent changes in modifications, each of which with potential impacts on wellbeing. The use of virtual platforms creates both physical and social separation, and act as “virtual walls” leading to a reduction in the quality and quantity of interactions between peers and instructors and feelings of isolation [27]. Also, using virtual learning for extended periods can lead to feelings of withdrawal and lowered motivation, particularly if students are not accustomed to, prepared for, or anticipated this learning style [28, 29]. Thus, it is essential to assess the mental health and wellbeing of students to provide supports that reflect student needs for effective learning and optimal motivation.

Full-time students in Malaysia have had to endure lengthy restrictions and educational modifications, potentially impacting well-being and mental health and in turn, affect the three basic psychological needs for intrinsic motivation [30–32]. Autonomy may be lost in students who feel a reduced ability to communicate with peers, instructors, and administrators. These are compounded by the immediacy of fluctuating restrictions, where time and logistics make it difficult for students to provide input. Also, since the virtual learning increases separation and isolation, it also reduces feelings of relatedness. Further, changes to expectations are known to dampen mental wellbeing and feelings of relatedness [33]. Finally, ineffective delivery of education, particularly in regions with technical challenges or for those who have limited digital literacy [34], may result in feelings of inadequacy or lowered perceived competence. Given that intrinsic motivation can be significantly impacted, there should be concern about the effects of low motivation, which may lead to attrition, burnout, or unprepared students.

The Malaysian context during the COVID-19 pandemic provides a unique opportunity to examine how a diverse group of undergraduate medical students have experienced the COVID-19 pandemic. This provides important insight about the shared and unique perspectives from various ethnographic populations. The goal of this study was to capture the perceptions of Malaysian medical students regarding impacts of the pandemic on their education and discern whether these experiences relate to major demographic variables. To do this, a questionnaire was designed with the intent to measure their perceived mental wellbeing and physical health risks, autonomy in decision making, perceived implications of distance learning on their education, and provide insight to changes that may affect graduation. An opportunity was also taken to assess the experiences and perceptions of clinical students about participation and safety in clinical postings during the COVID-19 pandemic. Here, the effects on student wellbeing and motivational profiles of the student population and within different subgroups are shown.

## Methods

### Study population

All undergraduate medical students actively registered in accredited medical schools within Malaysia were entitled to participate in the study, including both public and private institutions. Power estimates required that at least 341 responses were required to achieve a representative sample with confidence level of 95% and 0.05 margin of error, based on an estimated student population of 3,000 in Malaysia. Ethics approval was obtained from the Universiti Malaya Research Ethics Committee (UMREC) (UM.TNC2/UMREC_1241). Invitations to participate were distributed through the Society of Malaysian Medical Association Medical Students (SMMAMS) contacts with the accredited medical school student groups in Malaysia.

### Instrument design

An online cross-sectional survey was designed using the framework from the Association for Medical Education in Europe (AMEE) Guide No. 87 [35]. The survey was written in English, as most medical schools instruct only in English and others would use English bi-lingually. Specifically, items were developed using positive phrasing and likert-scales were used such that low values tended towards a negative alignment (*i.e.* “never”) and high values tended towards a positive alignment (*i.e.* “always”). The survey and composed of a demographic section, and seven sub-sections including those related to: wellness and concerns, experience with online learning, involvement in decision making, teaching and learning, assessment, graduation expectations, and a clinical-student section. Four items from the Patient Health Questionnaire-2 (PHQ-2) and Generalized Anxiety Disorder 2-item (GAD-2) screening tools for Major Depressive Disorder (MDD) and Generalized Anxiety Disorder (GAD), respectively [36, 37]. Other items were designed by the authors and reviewed by local experts. Items were selected to minimize length of the survey and to align with research objectives as piloting was not possible given time restraints.

### Statistical analysis

Data was prepared for analysis by removing incomplete surveys, if more than two subsections were incomplete, and items, if more than 10% of participants did not provide a response. Responses were codified using numerical values to represent variables. Statistical analysis was done using the IBM SPSS statistic package version 23.0. Items were validated using Principal Component Analysis (PCA) using varimax rotation. Validity was granted when the Kaiser-Meyer Olkin (KMO) was ≥ 0.70 and the Bartlett’s Test of sampling adequacy was significant (*p* ≤ 0.05) [38]. Components were selected if eigen-values were ≥ 1.0 (Campbell 2002). Items within the components required average communicality after extraction to be ≥ 0.60, given a sample size greater than 250 (Field 2009). Further, items required factor loading of ≥ 0.50 and those with cross-loadings ≤ 0.50 were excluded from analysis (Maskey 2018). Internal consistency of the survey was calculated using Cronbach’s α and considered sufficient if α ≥ 0.50 (Bowling 2009, Verma 2010). The resulting set of corrected items required the item-total correlation to be ≥ 0.20 and the leave-one-out Cronbach α coefficient to not be significantly changed (*i.e.* α ≤ 0.50) (Verma 2010).

Descriptive statistics were calculated for demographic variables and component scores. Component scores were calculated by averaging the responses within the component. Four items were reverse coded (PHQ and GAD items), which were inversed to calculate the component score. PHQ score and GAD scores were calculated as described previously [36, 37]. Data distribution was assessed for normality. T-Test and ANOVA calculations were used to assess whether responses were significantly different (*p* < 0.05) between two or more groups, respectively. Strength of correlations between demographic variables were determined using Pearson correlation coefficients (r^2^).

### Qualitative analysis

Respondents were invited to provide additional written comments, which were extracted and coded using thematic analysis. Thematic analysis was conducted in six steps: (1) the data was read several times to be familiarized with the data, (2) initial codes were generated for repeated or interesting comments, (3) initial codes were consolidated, and themes were identified, (4) the themes were reviewed and cross-checked with quoted comments, (5) the themes were named and defined, and (6) the themes were presented as findings of this study [39].

## Results

### Survey collection

Between March 23rd-June 3rd, 2021, 453 students responded representing 23 of 30 public and private accredited institutions across different states in Malaysia. Despite students from each institute having been invited through the SMMAMS, no responses were received from seven smaller institutions. However, since power estimates were met and there was representation from the majority of schools, the responses should be minimally biased. In total, 9 participant surveys and one item missing more than 5% of responses were removed from the study. Any remaining missing data was assessed pairwise.

### Demographics

The cohort of students represented a variety of demographic variables (Table 1). Students were self-assigned into pre-clinical (61.5% n = 273) and clinical (38.5% n = 171) stages of their undergraduate programmes. Females were represented by 66.3% of respondents and the average age was 20.84 (σ = 1.77). The ethnic profile of students represented Malay (28.2%), Chinese (53.5%), Indian (12.3%), and International (6%) cultures. Additionally, socio-economic status of students was measured using three cut points relating to tertiles of household income, specifically those below the 40th percentile (B40; 15.2%), within the middle 40th percentile (M40; 61.0%), and above the 20th percentile (T20; 23.8%). Demographic variables were not significantly correlated.

**Table 1:** Demographic variables

	n	Central tendency
Age	436	20.84 (1.77)*
*Stage*	444	
Preclinical	273	61.5%
Clinical	171	38.5%
*Gender*	433	
Male	144	33.3%
Female	287	66.3%
Non-binary	2	0.5%
*Ethnicity*	415	
Malay	117	28.2%
Chinese	222	53.5%
Indian	51	12.3%
International	25	6.0%
*Income*	447	
Below 40th Percentile	65	15.2%
Middle 40th Percentile	261	61.0%
Top 20th Percentile	102	23.8%

*Mean

### Components

Demographic, categorical, tick-box, and qualitative items were removed for PCA. Convergence between the remaining 40 items was met after 4 iterative rounds of PCA, resulting in eight components comprising 32 items in total (KMO = 0.802, *p* < 0.000). Internal consistency was estimated for all items combined (α = 0.622) and using leave-one-out analysis of each item (α ≥ 0.50 for all). The items within the resulting components were thematically related to: Mental Health, Depth of Learning, Social Concern, Communication, Timing, Delayed Graduation, Assignments, and Student-Centred Learning (Table 2). The dataset that includes average responses for each item within the components, which support the conclusions of this article are available in Additional file 1.

**Table 2:** Average component scores by selected demographic variables

		Mental health	Depth of learning	Concern	Communication	Timing	Graduation	Virtual Assessment	Student-Centred Learning
		C1	C2	C3	C4	C5	C6	C8	C9
All students	Mean	2.81	2.18	3.02	2.58	3.47	3.23	2.66	3.34
	SD	0.80	0.65	0.90	0.76	0.57	1.10	1.44	0.80
*Stage*									
Pre-clinical	Mean	2.82	2.26*	3.03	2.60	3.39*	3.07*	2.73	3.20*
	SD	0.76	0.63	0.85	0.78	0.49	1.08	1.53	0.74
Clinical	Mean	2.88	2.06*	3.00	2.57	3.60*	3.47*	2.56	3.55*
	SD	0.86	0.65	0.98	0.74	0.65	1.09	1.27	0.86
	*p*		0.002			0.000	0.000		0.000
*Gender*^a^									
Male	Mean	3.10*	2.27	2.93	2.51	3.41	3.07	2.71	3.50*
	SD	0.71	0.65	0.84	0.73	0.57	1.10	1.48	0.78
Female	Mean	2.73*	2.14	3.06	2.63	3.49	3.28	2.59	3.26*
	SD	0.82	0.64	0.93	0.78	0.56	1.10	1.40	0.81
	*p*	0.000							0.013
*Ethnicity*									
Malay	Mean	2.60*	2.10	3.11	2.68	3.40	3.35*	2.80	3.30
	SD	0.78	0.67	0.86	0.72	0.57	0.99	1.39	0.78
Chinese	Mean	3.05*	2.23	2.93	2.59	3.48	3.25*	2.50	3.36
	SD	0.73	0.57	0.91	0.79	0.55	1.10	1.41	0.81
Indian	Mean	2.74*	2.34	3.12	2.45	3.51	2.75*	2.71	3.33
	SD	0.82	0.86	0.89	0.60	0.66	1.14	1.61	0.80
International	Mean	2.32*	2.01	3.21	2.36	3.51	3.11*	2.44	3.50
	SD	0.93	0.73	0.96	0.95	0.55	1.32	1.39	0.76
	*p*	0.000					0.013		
*Income*									
B40	Mean	2.77	2.10	3.14*	2.56	3.46	3.33	2.70	3.30
	SD	0.90	0.57	0.92	0.63	0.50	1.07	1.47	0.95
M40	Mean	2.86	2.17	3.05*	2.51	3.46	3.29	2.74	3.36
	SD	0.79	0.69	0.90	0.77	0.59	1.13	1.46	0.80
T20	Mean	2.82	2.21	2.80*	2.71	3.49	3.07	2.49	3.29
	SD	0.77	0.58	0.87	0.80	0.55	1.02	1.37	0.76
	*p*			0.026					

SD, Standard Deviation; B40, Bottom 40th percentile; M40, Middle 40th percentile; T20, Top 20th percentile; C, Component

*Indicate mean scores that are significantly different from at least one other variable (*p* < 0.05)

^a^Non-binary students excluded due to the small sample size

### Wellbeing

**Fig. 1:**
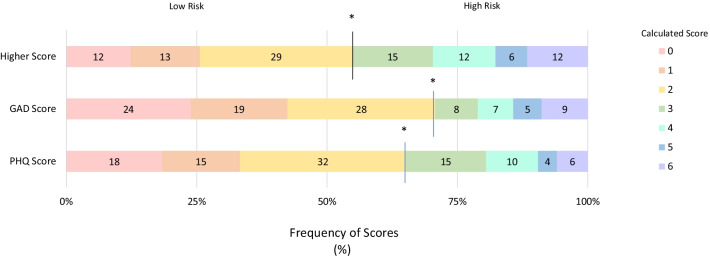
Distribution of PHQ and GAD scores amongst all students, given in percent of total student population. Combined higher scores indicate total number of students at risk of MDD or GAD, specifically those with either GAD or PHQ score ≥ 3. Risk threshold is indicated by the asterisk (*)

Student mental health was assessed using the PHQ-2 and GAD-2 items, which indicated that 45% of students were likely to be experiencing MDD or GAD (Fig. 1). These were captured in Component 1, which reflected responses from students reporting little interest, feeling depressed, or the inability to control worrying for at least several days in the prior two weeks (Table 2). Female students were more likely to respond negatively overall towards their mental health (*p* < 0.000) (Table 2) and were at a significantly higher risk of MDD and GAD (*p* < 0.000) (Table 3). Ethnicity was also related to mental health, as Chinese students indicated having better mental health compared to the remaining students' average (*p* < 0.000) (Table 1). Additionally, students provided qualitative comments regarding concerns about their own or their peers mental health (Table 4).

**Table 3:** Selected item descriptors by subgroup

Grouping	Item	Variable	Mean	SD	*p*
All students	How much have students been able to contribute to modifications in the medical program during the COVID-19 pandemic?	–	2.48	1.03	–
	Do you think program administrators consider your overall well-being when considering modifications to the medical program?	–	2.44	0.98	–
	Do you consciously worry about your peers/instructors developing COVID-19?	–	3.03	1.18	–
	Do you feel that you are being prepared for your profession?	–	2.00	0.88	–
	Do you feel that virtual assessments accurately measure your theoretical knowledge?	–	3.02	0.98	–
	Do you feel that virtual assessments accurately measure your clinical skills?	–	1.94	0.93	–
	Do you feel that virtual assessments accurately measure your professionalism?	–	2.28	1.03	–
	Concerned about a delays on preparedness	–	2.94	1.32	–
	Do you consciously worry about your family developing COVID–19?	–	3.84	1.15	–
Stage	What percent of time have you spent using online learning environments since March 2020?	Pre-Clinical	4.72	0.76	0.000
		Clinical	4.40	0.92	
	Are you concerned about a delayed graduation on your preparedness for the profession?	Pre-Clinical	2.76	1.31	0.000
		Clinical	3.22	1.29	
	Do you think program administrators consider your overall well-being when considering modifications to the medical program?	Pre-Clinical	2.53	1.03	0.015
		Clinical	2.29	0.88	
	Overall, are you satisfied with the delivery of medical education during the COVID-19 pandemic?	Pre-Clinical	2.45	0.97	0.004
		Clinical	2.17	0.98	
	Are you concerned about your ability to secure housemanship after graduation based on modifications to your programming made during the pandemic?	Pre-Clinical	3.38	1.18	0.003
		Clinical	3.72	1.19	
	PHQ2 Score	Pre-Clinical	2.31	1.55	0.039
		Clinical	1.98	1.75	
	Do you feel that virtual assessments accurately measure your theoretical knowledge?	Pre-Clinical	3.12	0.095	0.006
		Clinical	2.85	1.06	
	Do you feel that virtual assessments accurately measure your clinical skills?	Pre-Clinical	2.06	0.94	0.000
		Clinical	1.74	0.89	
	Do you feel that virtual assessments accurately measure your professionalism?	Pre-Clinical	2.37	1.006	0.026
		Clinical	2.15	1.04	
Gender	Are you concerned about a delayed graduation on your preparedness for the profession?	Male	2.69	1.29	0.008
		Female	3.05	1.32	
	PHQ2 Score	Male	1.74	1.42	0.000
		Female	2.37	1.69	
	GAD2 Score	Male	1.42	1.56	0.000
		Female	2.36	1.86	
	Would you like the teaching to be more interactive?	Male	3.72	1.03	0.006
		Female	3.38	1.08	
Ethnicity	Do you consciously worry about your family developing COVID-19?	Malay	4.08	1.02	0.004
		Chinese	3.65	1.19	
		Indian	3.96	1.02	
		International	4.08	1.19	
	Do you feel that you are being prepared for your profession?	Malay	1.89	0.95	0.000
		Chinese	1.92	0.77	
		Indian	2.54	0.95	
		International	2.20	1.00	
	Are you able to communicate concerns about SOPs to coordinators (or those overseeing your postings)?	Malay	3.19	1.02	0.004
		Chinese	2.99	1.18	
		Indian	2.13	0.72	
		International	2.50	1.03	
	PHQ2 Score	Malay	2.55	1.65	0.001
		Chinese	1.92	1.51	
		Indian	2.24	1.66	
		International	2.96	1.99	
Income	Are you concerned about the direct financial impact of the pandemic on you or your family?	B40	3.69	1.27	0.000
		M40	3.41	1.28	
		T20	2.84	1.30	
	Do you receive feedback for improvement from your instructors currently, during the pandemic?	B40	2.66	0.91	0.019
		M40	2.70	1.05	
		T20	3.02	1.04

**Table 4:** Representative qualitative student responses

Theme	Response
Mental Health	“At the end of the day, a lot of the students concerns esp[ecially] mental health are disregarded and dismissed as something we SHOULD be able to adapt and cope with”
COVID-19 Concerns	“I feel like medical students' heath was neglected as the clinical practice was still ongoing despite the spike in the cases in Malaysia. I understand the importance of completing the course as soon as possible but somehow we are exposed to a greater risk of being infected”
Communication	“Students always try their best to propose suggestions but somehow they are not being considered” “Even if considerate involvement exists in the system…no further action is taken” “Should emphasis on every medical student, but not only consider those clinical med student”
Clinical students	“The clinical skills learning is greatly impacted by the SOPs. Postponed clinical sessions has led to deteriorating skills, and the current SOPs has made clinical skills learning near impossible…” “…I finished the entire internal medicine posting in online… I personally have a feel[ing] that I'm not going to be a competent enough in clinical skills when I'm graduating…”

Student wellbeing was also assessed by asking students about their concerns and worries in factors related to contracting COVID-19 and its transmission to self, peers or instructors, or family. Students mostly reported minimal to moderate concern, which related to their physical wellbeing if they were to be infected with SARS-CoV-2 or in working at clinical environments (Component 3; Table 2). Interestingly, most students expressed high levels of worry regarding peers or instructors developing COVID-19 (µ = 3.03, σ = 1.18) (Table 3). Concern was also significantly associated with income, as those in lower income brackets indicated more concern overall (*p* = 0.026) (Table 2) and with regards to financial impact of the pandemic (*p* < 0.000) (Table 3). Within Component 3, ethnicity was related to concern about family members developing COVID-19 (*p* = 0.004) (Table 3).

### Learning experiences

Learning experiences were reflected in the responses pertaining to depth of learning (Component 2.), and modifications related to timing (Component 5) (Table 2). Most students were satisfied with the depth of learning, based on responses about accuracy of virtual theoretical assessments and perceptions of their learning overall (Component 2) (Table 2). However, students felt that clinical skills and professionalism were less accurately assessed using virtual tools (µ = 1.94, σ = 0.93; µ = 2.28, σ = 1.03) (Table 3). Interestingly, pre-clinical students were more likely to feel that virtual assessments mostly or always measured theoretical knowledge accurately (*p* = 0.006), similar in trends about clinical skills and professionalism (*p* < 0.000, *p* = 0.026) (Table 3). Also, pre-clinical students were more satisfied with the delivery of their medical education during the pandemic than their clinical counterparts (μ = 2.45, 2.17; *p* = 0.004) (Table 3). Timing, which included amount of time spent using virtual platforms and changes to graduation, had greater effects on pre-clinical and clinical students, respectively (Component 5, *p* < 0.000) (Table 2), particularly since pre-clinical students spent more time using virtual learning environments (*p* < 0.000) (Table 3).

The amount of interactivity and ability to ask questions in class was reflected by the student-centred learning component (Component 9), such that most students indicated that they could minimally or never ask questions and that they would like to have more interactive teaching (Table 2). Pre-clinical students experienced less student-centred learning than clinical students (*p* < 0.000) (Table 2).

### Future readiness

Preparedness arose in the components related to depth of learning (Component 2) and graduation (Component 6). On average, students felt that they were only being prepared ‘a little bit’ for their profession (μ = 2.00; σ = 0.880) and clinical students felt significantly less prepared based on changes to graduation (Component 6) (Table 2); (*p* < 0.000) (Table 3). Indian students felt that they were being more prepared for their profession (*p* = 0.000), while female students felt that changes made to graduation would result in less preparedness, compared than their respective counterparts (*p* = 0.008) (Table 3). Further, students indicated some concern about effects of delayed graduation on preparedness, but pre-clinical students indicated less concern about securing horsemanship based on delayed graduation than clinical students (*p* < 0.003) (Table 3).

### Communication

Students reported to receive limited communication (Component 4), which included a lack of feedback from instructors (Table 2). Specifically, income was related to perceived levels of feedback, as those in lower income brackets indicated receiving less feedback (*p* = 0.019) (Table 3). Also, students reported to have been moderately consulted about modifications made to programming during the pandemic and students indicate that their wellbeing was being adequately considered during decision making (Table 3). Additionally, more pre-clinical students felt that their wellbeing was being considered in decision making compared to clinical students (*p* = 0.015) (Table 3). A number of students provided comments regarding a lack of consideration or inclusion in the decision making process (Table 4).

### Clinical subsection

**Fig. 2:**
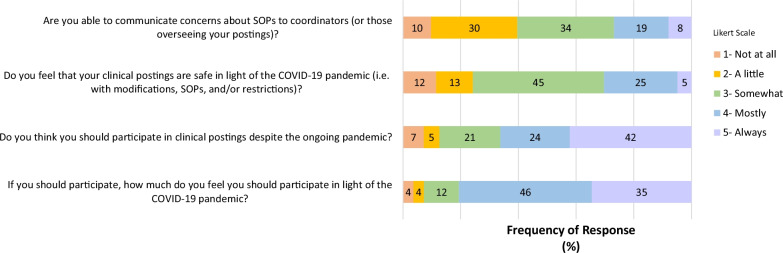
Clinical student responses indicate some concern regarding ability to communicate safety concerns, but an overall inclination towards participation

Clinical students responded to items about communication, safety, and participation in their clinical postings. Most clinical students felt they were at least sometimes able to communicate concerns about SOPs with coordinators (Fig. 2). Differences existed in the degree of ability to communicate based on ethnicity (*p* = 0.004) (Table 3). Importantly, 45% of students felt that there were safety concerns in their clinical postings, though a majority of students indicated that they should at least mostly participate (Fig. 2). Some students commented about the effects of SOPs on the quality of their clinical postings (Table 4).

### Qualitative findings

There were 83 participants who provided optional comments that were used for thematic analysis. Three major themes were identified including: student concerns for overall health (Wellness), challenges with student involvement in medical curricula modifications (Communication), and interruptions to the clinical portion of the MBBS programmes (Clinical Posting Interruptions) (Table 4).

## Discussion

The COVID-19 pandemic affected medical schools worldwide, requiring schools to respond quickly by adapting to virtual learning environments and by modifying clinical placements. Medical students were expected to manage these changes in addition to the universal challenges of a global crisis, giving them a unique perspective during the pandemic. The population of Malaysia includes three major ethnicities and a broad distribution of socioeconomic groupings, which is reflected by the medical student population. By surveying all medical students in Malaysia, it was possible to characterize the perspectives shared by students nationally and those which may be related to specific ethnographic groups. The results indicate that student perceptions converge on eight domains relating to well-being, depth of learning experience, and communication. Interestingly, none of the components included items relating to technical delivery of virtual learning, suggesting that any technical challenges were independent of the pandemic-related experiences of students and are not an underlying factor in student well-being or motivation [13, 20].

The components identified in this study align with self-determination theory and help to understand the intrinsic motivation of students during the pandemic. Of concern, a large percent of students met the risk threshold for experiencing MDD or GAD. This was supported by claims about having depressed or anxious symptoms and experiencing considerate, or even overwhelming, concern and was validated through student comments. While baseline mental health and wellness are unknown, students did comment that their mental health has been negatively affected by the pandemic, as seen elsewhere [20, 40, 41]. Importantly, levels of mental health and wellbeing, which are intricately related to each psychological need, suggesting that motivation may also be low [42]. Results like these have been of concern, as the reduction in mental health will also affect both student learning and personal satisfaction [30, 43, 44]. Indeed, strategies to improve student well-being and maintain motivation are being developed to specifically target students emotionally affected by pandemic-related restrictions [45].

While well-being may affect each motivational need, other components corresponded to specific needs. Communication, for example, is required for relatedness and autonomy, as the interaction with others can promote emotional connectedness and independence, respectively [46, 47]. Communication is also integral to education, making it an important measure of the effectiveness of instruction and experiences of students. Here, students provided negative sentiments about engagement in the classroom and formative and summative assessment, suggesting that communication with instructors has been impeded and may indicate low feelings of relatedness [48]. Similarly, while students some consultation in decision making about pandemic-related modifications, numerous comments regarding the student role, or lack thereof, in decision-making processes, suggesting that student choice may not have been implemented. This overall reduction in communication suggests that students feel that they are not being validated, subsequently leading to reduced autonomy and motivation [49]. This is supported by reported evidence that positive relationships with instructors and being provided choice are related to feelings of autonomy [47, 50, 51]. Unfortunately, these opportunities were limited in Malaysia given the need to respond to frequent changes in COVID-19-restrictions, making it difficult to establish relationships or effectively consider student feedback [52]. These results show that better communication strategies should be prioritized to improve student engagement in the classroom and incorporate student choice to promote relatedness and autonomy.

Developing competence is a priority to educators, given that the ultimate learning objectives of any programme are to develop student competencies. However, competence is also a component of self-determination and is intrinsic to the learning process, since only someone who is convinced about their ability to accomplish a task would be motivated to attempt the task [30, 31]. Here, perceptions about depth of learning and preparedness provided insight to students’ self-perceived competence during the pandemic. Importantly, students indicated an overall satisfaction with their MBBS programmes and that the quality of their education has not been greatly changed. This suggests that there must be some degree of competence felt by the students. Comparatively, students responded that they are being under prepared for their profession and exhibited negative feelings towards formative feedback and the accuracy of some virtual assessments. These perceptions may relate to low self-confidence and difficulty interpreting their standings, likely leading to reduced feelings of competence [42, 48]. This may have arisen since guidance to help students better understand their progression, which requires a social context, has likely been hindered during the pandemic [53–55]. Taken with the overall student satisfaction, competence in students likely exists, but may not be self-evident resulting in lowered motivation.

The self-determination profile of Malaysian medical students, as evidenced by the negative perceptions towards relatedness, autonomy, and competence, indicate an overall reduction of intrinsic motivation during the COVID-19 pandemic. More work is needed to determine the implications of these findings, but indicate that supports should be considered for each of the psychological needs to holistically mitigate the impacts of covid-related modifications on student motivation [49]. Further, as the pandemic resolves, student experiences will carryforward, impacting future learning and success if negative experiences are not remedied.

Perceptions about the COVID-19 pandemic were also evaluated for trends in sub-populations of students, with notable differences occurring between pre-clinical and clinical stages. While overall mental health and concern was similar between pre-clinical and clinical students, their learning experiences varied greatly. This is most attributed to the need for specific modifications from the different style of programme delivery for each group [15, 56, 57]. In Malaysia, preclinical students were transitioned to a virtual learning space, providing consistent, if not preferred, learning opportunities. This consistency may be why pre-clinical students more often felt that their overall well-being was being considered in the decision making of modifications. In contrast, pre-clinical students showed higher risk of depression and indicated that instructional delivery was less student-centered compared to clinical students. These perceptions may stem from the virtual wall if students have felt forgotten or isolated and may imply a reduction in autonomy and relatedness [20, 49, 50]. Conversely, clinical students were more affected by timing modifications, likely due to frequent changes to time, depth, or quality of clinical placements, and which have resulted in less satisfaction towards modifications made during the pandemic. The disruption to clinical teaching may also relate to their greater feelings of unpreparedness and concerns about securing housemanship positions, indicating a lowered sense of competency and to some degree of autonomy [15, 21, 58]. However, clinical students generally supported participation in clinical postings during the COVID-19 pandemic, and though some safety concerns or the ability to communicate their concerns were evident, this suggests that any reductions in motivation did not undermine the desire to contribute [59]. Different in experiences of pre-clinical and clinical students are expected, so characterizing them is useful to identify the specific needs of each group.

The diversity of students represented by the Malaysian medical student body provides an opportunity to explore whether there are ethnographic contributors to perceptions on educational experiences during the pandemic. This is of particular interest as the fundamental concepts of self-determination theory are universal, though there is less known about motivational profiles between demographic variables, particularly during the pandemic [42, 60]. Here, gender was related to the largest difference in experiences between students during the pandemic. Female students exhibited significantly worse mental health, perceived less depth of learning and student centredness, and were more concerned about delays on preparedness, implying reduced levels of intrinsic motivation. Since a recent Malaysian study reported no significant difference in online learning readiness during the pandemic between male and female students [61], these differences are not likely due to capabilities. Interestingly, prior to the pandemic, women have consistently expressed have a more self-determined profile than male counterparts, so it is curious which aspects of the pandemic-experience have contributed to these differences [32]. Given the similar life-stage of medical students in Malaysia, it is more likely that these feelings may arise from deeper internalization of concern, loneliness, and social separation, particularly given the striking difference in mental health [62–64]. Also, it is possible that female students who had returned home for virtual learning took on more household responsibilities. Globally, women have been disproportionately burdened with these responsibilities during the pandemic, which has attributed to different emotional responses of the genders in general and in learner groups [62, 64, 65]. The disparities between gender needs should be particularly highlighted during challenging times to provide appropriate support and ensure existing systemic biases are not exacerbated.

Ethnicity was also related to perceived experiences and motivational profiles of Malaysian medical students during the pandemic. The biggest difference arose in mental health and the impacts of change made to graduation. Interestingly, compared to their peers, Chinese students reported better mental health and were less concerned about family members developing COVID-19, perhaps indicating greater confidence during the pandemic. International students reported the lowest levels of mental health, which may be related to feelings of isolation if they are distanced from their native community or culture, since they also reported more concern about family developing COVID-19. Interestingly, ethnicity was also related to perceptions about modifications made to graduation, as Indian students were least concerned about the impact of any delay. Attributing these findings to specific cultural norms would be inaccurate and insensitive in this context [60], but does indicate the need to ensure that all students, regardless of ethnicity, are properly supported, particularly in populations with such diverse representation.

Malaysian medical students from each income bracket are entitled to enter MBBS programmes through various streams including affordable, yet competitive, public universities, various funding initiatives, or more costly private universities [66]. Here, student groupings from different income brackets were found to have significantly different levels of concern, specifically those in the lowest income bracket who reported more concern about financial impacts from the pandemic. This may indicate a lower level of relatedness and autonomy, particularly if other students do not share the same concern or if additional costs may be incurred for virtual learning. They also indicated receiving less formative feedback, which may be related to technical issues. Indeed, it has been reported that, in addition to the fact that students in remote areas have faced technical challenges during remote learning, the economic impacts of the pandemic have disproportionately affected people in lower income groups [17, 65]. Indeed, the availability of technical devises and internet has been inversely correlated to COVID-19 related anxiety [65]. Equity in education is essential and students from lower income bracket might need additional financial support in securing equal learning opportunities during the COVID-19 pandemic [13, 67]. It is thought that improving accessibility to virtual resources may also improve mental health, which may in turn improve motivation of these students [65]. While alleviating financial constraints can be challenging, it is understood that institutions are trying to distribute resources to affected students, which would improve accessibility as well as helping students to feel validated.

Characterizing the experiences of medical students during the COVID-19 pandemic will help to develop inclusive strategies meant to mitigate any long-term impact caused by the pandemic-related modifications made to undergraduate medical education. This will, of course, require accommodation of diverse student needs and recognition that needs may be complicated by co-variates. While the scope of this study excludes a comprehensive analysis of how demographic features interact, we did find some demographic variances within subgroups. For example, Malay students in clinical stages expressed that they received more formative feedback and were more confident in their ability to voice safety concerns, which may relate to higher feelings of autonomy, despite average inclinations of students towards reduced communication during the pandemic. Some other differences were also seen within components of the survey, like feelings of preparedness or concerns about securing future positions, but more work is needed to better interpret these findings.

## Conclusions

Taken together, these findings show how modifications made to medical education during the COVID-19 pandemic have impacted students, particularly in relation to mental health and other factors relating to self-determination. Overall, students expressed a lower level of motivation, as relatedness, autonomy, and competence were all affected by the changes. Within the Malaysian undergraduate medical student population, it is evident that student and demographic groups have experienced the pandemic differently, resulting in unique motivational profiles. Analyzing student experience is necessary to recognize how students have been uniquely affected and how educators can support them as they progress through their education and into the medical profession. Additionally, understanding the state of student well-being, experiences with learning, and ability to communicate during the pandemic, will help to better scaffold student learning. Ultimately, this study indicates that medical students across Malaysia have experienced relatively lower levels of self-determination during the COVID-19 pandemic.

## Supplementary Information

**Additional file 1**. Student responses for the valid items that comprise the components of the survey.

## Data Availability

The datasets used and/or analysed during the current study are available from the corresponding author on reasonable request.

## Abbreviations

SOP: Standard operating procedure
GAD: Generalized anxiety disorder
MDD: Major depressive disorder
SMMAMS: Society of Malaysian Medical Association Medical Students
AMEE: Association for Medical Education in Europe
C: Component
KMO: Kaiser–Meyer Olkin
MBBS: Bachelors of medicine and bachelor of surgery
MQA: Malaysian Qualifying Agency
MMC: Malaysian Medical Council
